# Periodontal Status of Patients with Celiac Disease and Non-Celiac Gluten Sensitivity: A Literature Review

**DOI:** 10.3390/jcm15082828

**Published:** 2026-04-08

**Authors:** Thaleia Angelopoulou, Yiorgos A. Bobetsis

**Affiliations:** 1 School of Medicine, National and Kapodistrian University of Athens, 11527 Athens, Greece; thangelop@yahoo.gr; 2 Department of Periodontology, School of Dentistry, National and Kapodistrian University of Athens, 11527 Athens, Greece

**Keywords:** periodontal diseases, periodontitis, gingivitis, oral health, celiac disease, non-celiac gluten sensitivity, gluten-related disorders, gluten intolerance, gluten-free diet

## Abstract

**Background/Objectives**: Celiac disease (CD) is a chronic, immune-mediated enteropathy induced by dietary gluten exposure in genetically predisposed individuals. Along with non-celiac gluten sensitivity (NCGS), these disorders present with multiple intestinal and extra-intestinal symptoms leading to multisystemic involvement, with complications documented in the oral cavity as well. Persistent immune activation and dysregulation, chronic inflammation, nutrient deficiencies, xerostomia, and microbial dysbiosis found in CD and NCGS constitute shared pathological findings, providing biological plausibility for an association with periodontitis. **Methods**: A narrative literature review was conducted based on a systematic search of four databases (PubMed, Scopus, Web of Science, Cochrane Library) and the gray literature through January 2026. A comprehensive set of clinical, radiographic, biochemical and immunological parameters was assessed. Two reviewers independently screened and selected studies, with disagreements resolved by consensus. **Results**: A total of 15 studies met the eligibility criteria and were included in the review. Available evidence, mainly derived from cross-sectional observational studies, remains limited, methodologically heterogeneous, and largely inconclusive. Across adult and pediatric populations, findings do not consistently demonstrate a clinically meaningful association between CD or NCGS and periodontal inflammation, irrespective of gluten-free diet (GFD) adherence. Observed differences, when reported, are modest and inconsistent, and can be mainly attributed to oral hygiene behaviors and dental visit patterns. **Conclusions**: Despite considerable biological plausibility linking gluten-related disorders with periodontal inflammation, current evidence does not support a definitive conclusion regarding the impact of CD or NCGS on periodontal health.

## 1. Introduction

Celiac disease (CD) is a chronic immune-mediated enteropathy triggered by dietary gluten in genetically susceptible individuals [1,2]. Its global prevalence is currently estimated to range from 0.7% to 1.4%, depending on the diagnostic criteria employed, and accumulating epidemiological evidence indicates a steady increase in incidence worldwide, underscoring its growing public health significance [3,4]. This upward trend is likely attributable to the broader use of more sensitive diagnostic approaches, heightened disease awareness, and possible shifts in modern dietary patterns, including increased consumption of wheat, rye, and gluten-containing products compared with past dietary habits [5,6].

The clinical manifestations of CD are highly heterogeneous, ranging from mild to severe intestinal and extra-intestinal symptoms; patients may also present with non-classical manifestations or be entirely asymptomatic, with many such cases often remaining undiagnosed [7,8,9,10,11]. From a pathophysiological perspective, CD is characterized by a progressive immune-mediated inflammatory response of the small intestinal mucosa, predominantly affecting the duodenum, resulting in villous atrophy and subsequently, impaired nutrient absorption [12,13]. Persistent immune activation and chronic inflammation may extend beyond the gastrointestinal tract, leading to multisystemic involvement and a broad range of extra-intestinal complications, including skeletal, reproductive, cardiovascular, neurological, and psychiatric complications [5,8,14]. Untreated CD has also been associated with an increased risk of adverse long-term outcomes, such as intestinal adenocarcinoma, enteropathy-associated T-cell lymphoma, and other forms of non-Hodgkin lymphoma [14,15,16].

The majority of patients with CD carry the HLA-DQ2 and/or HLA-DQ8 haplotypes, the absence of which has a high negative predictive value and effectively excludes the diagnosis in most cases [17,18]. According to current guidelines, diagnosis is based on celiac-specific serological testing, including the detection of IgA anti-tissue transglutaminase (anti-tTG) and anti-endomysial (anti-EMA) antibodies, in combination with biopsy-confirmed duodenal villous atrophy [2,19,20,21]. Histopathological findings are characterized by intraepithelial lymphocytosis and crypt hyperplasia and are graded according to the Marsh–Oberhuber classification, in which higher grades correspond to increasing disease severity [22,23,24].

Clinical symptoms in individuals affected by CD often regress following adherence to a strict gluten-free diet (GFD) [1,2,25,26]. However, this response is not consistent in all patients [27,28,29,30,31]. Even though lifelong adherence to a GFD is the standard disease management strategy, compliance remains challenging due to the widespread presence of gluten in food products, the risk of inadvertent cross-contamination, and the inferior nutritional quality of gluten-free alternatives [12,32,33,34,35].

In addition to CD, non-celiac gluten sensitivity (NCGS) has been recognized as another gluten-related disorder characterized by intestinal and extra-intestinal symptoms triggered by gluten ingestion in individuals who do not fulfill the diagnostic criteria for CD or wheat allergy (WA) [36,37,38,39]. NCGS is currently diagnosed as presence of symptoms following gluten exposure in combination with clinical improvement after GFD [40,41,42,43], in the absence of celiac-specific serology and immune-mediated villous atrophy [36,44,45,46,47,48,49], while HLA-DQ2 and HLA-DQ8 haplotypes are detected in a subset of patients at frequencies slightly exceeding those of the general population and histological alterations, if present, are typically mild [46,50,51,52,53,54]. Although CD and NCGS share overlapping clinical manifestations, they are typically classified as separate conditions, with no clear consensus on their distinction [36,37,52,55,56]. Emerging evidence suggests that NCGS is primarily associated with activation of innate immune pathways [36,41,50,57], increased intestinal permeability [50,58], and alterations in gut microbial composition [40,58], supporting partially shared mechanisms with CD.

Both CD and NCGS have been associated with a range of oral manifestations, including enamel defects, delayed dental eruption, xerostomia, and recurrent aphthous lesions [59,60,61,62]. Periodontal conditions, such as gingivitis and periodontitis, have more recently been investigated for potential associations with gluten-related disorders, particularly CD; however, the available evidence remains scarce and does not allow definitive conclusions. Hence, the influence of GFD adherence on periodontal outcomes has not yet been clearly defined. To date, no review has specifically addressed this topic. Therefore, the aim of the present review was to assess the periodontal status of individuals with CD and NCGS.

## 2. Materials and Methods

### 2.1. Study Design

A comprehensive narrative literature review was performed to explore the relationship between CD and NCGS and periodontal status and to evaluate the potential impact of adherence to a GFD on periodontal health in affected individuals. This literature review was conducted in accordance with the quality assessment criteria defined by the Scale for the Assessment of Narrative Review Articles (SANRA) guidelines [63]. The SANRA framework was used to ensure clarity of the aim, justification of the topic, adequate description of the literature search, appropriate referencing, and coherent scientific reasoning.

### 2.2. Information Sources and Search Strategy

A systematic search was performed and completed through 10 January 2026, to ensure that the available literature on the relationship between gluten-related disorders and periodontal status was comprehensively included in this review. Database-specific strategies were developed for PubMed, Web of Science, Scopus and Cochrane Library. A partial gray literature search was conducted using BASE, ProQuest, ResearchGate, and Google Scholar (Table S1). Studies were collected and exported to Mendeley Reference Manager (version 2.135.0), and duplicate hits were removed; duplicates not identified by the software were manually removed. Articles were selected based on their contribution to the understanding of the relationship between CD, NCGS, and periodontal status, in line with the scope of this literature review.

### 2.3. Eligibility Criteria

The selection of studies was conducted in accordance with a predefined set of inclusion and exclusion criteria established prior to the initiation of the review process. Eligible studies included prospective clinical studies, randomized controlled trials, case-control studies, cross-sectional studies, and cohort studies assessing the association between CD and NCGS and periodontal health. Included studies were required to involve human participants and to clearly describe the diagnostic criteria used to define the gluten-related disorder. Only peer-reviewed articles published in or translated into the English language were considered. Studies with designs different from those specified in the inclusion criteria, including systematic reviews, narrative reviews, letters, editorials, commentaries, case reports, as well as in vitro and animal studies, were excluded from the review.

### 2.4. Study Selection

**Figure 1:**
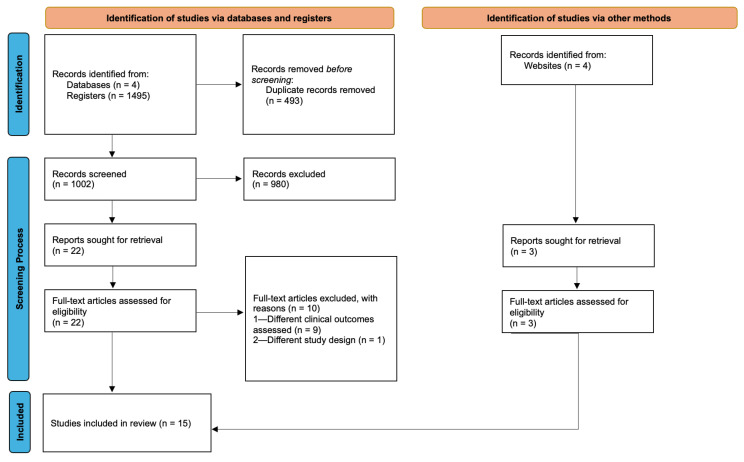
Flow diagram of literature search and selection process.

Study selection comprised two phases. Independent screening of titles and abstracts by both reviewers was followed by full-text assessment of potentially eligible reports. The reference lists of the included studies were also screened by one author (T.A.) to identify additional eligible trials. Discrepancies in selected articles were resolved through discussion until consensus was reached between the two reviewers. Inter-reviewer agreement during screening was evaluated using Cohen’s kappa coefficient [64]. A total of 15 studies met the inclusion criteria and were included in the review. The entire selection process is illustrated in Figure 1. Detailed information about excluded articles and the reasons for exclusion are provided in Table S2.

### 2.5. Data Collection Process, Data Items and Synthesis of Results

The two reviewers independently extracted data from the included studies using a standardized data extraction form developed in a Microsoft Excel spreadsheet. The following information was collected: publication details (authors, country, year, journal, language), study design, sample size and participant characteristics (including demographics, periodontal status-related parameters, and gluten-related disorder status and diagnostic criteria). Findings from the included studies were synthesized descriptively to summarize the available evidence. Given the narrative design of the review, a risk-of-bias assessment was not performed; instead, methodological limitations were considered narratively during study appraisal and synthesis.

## 3. Periodontal Status in Patients with Gluten-Related Disorders

A limited number of studies have investigated the impact of gluten-related disorders on periodontal status. In this review, a total of 1495 records were identified through database searching, with three additional records identified through gray literature searching. After duplicate removal and screening of titles/abstracts, 25 studies underwent full-text assessment. In this second phase, 10 studies were excluded due to different clinical outcomes assessed (n = 9) or different study designs (n = 1) (Table S2). Overall, 15 studies met the eligibility criteria and were included in this review. Among them, six studies evaluated clinical periodontal indices and oral hygiene parameters in adult populations, while seven studies focused on pediatric populations. In addition, two studies investigated radiographic findings related to alveolar bone loss, and three trials examined biochemical or immunological parameters related to periodontal inflammation, including salivary antimicrobial peptides and inflammatory biomarkers.

### 3.1. Clinical Periodontal Indices and Oral Hygiene Parameters

#### 3.1.1. Adults

In a cross-sectional questionnaire-based study, van Gils et al. [65] evaluated 980 participants, including 740 patients with biopsy- and/or serology-confirmed CD and 279 healthy controls. All participants with CD reported adherence to a GFD; 9% acknowledged partial non-adherence, and 5% of healthy controls also reported following a GFD despite the absence of CD. The frequency of dental visits per year did not differ between groups; however, self-reported gingival problems were significantly more frequent among patients with CD (*p* < 0.01).

In a cross-sectional analysis of the NHANES 2009–2012 data, Spinell et al. [66] evaluated 6661 adults and reported an overall CD prevalence of 1.08% (n = 49). Disease diagnosis was based on positive serological testing for anti-tissue transglutaminase (anti-tTG) and anti-endomysial (anti-EMA) antibodies. Of these participants, 15 were already diagnosed with CD, whereas 34 were identified as having undiagnosed CD through serological testing performed within the context of the study, indicating that most affected individuals were unaware of their condition prior to participation and thus, were not adhering to a GFD as treatment. The prevalence of moderate/severe periodontitis, as defined by the CDC/AAP criteria [67], was 40%, while the combined prevalence of diagnosed and undiagnosed celiac disease was 0.74%. Mean clinical attachment loss (CAL) tended to be lower among individuals with CD; however, these differences were not statistically significant. Probing pocket depth (PPD) values were significantly lower among both diagnosed and undiagnosed CD patients compared with healthy participants (*p* = 0.03), with similar findings observed in the proportion of sites with PPD ≥ 4 mm (*p* = 0.02). Although mean tooth loss values were numerically higher among participants with diagnosed and undiagnosed celiac disease, these differences were not statistically significant after multivariable adjustment (*p* = 0.72). Participants with diagnosed CD tended to floss more frequently than healthy and undiagnosed individuals (*p* = 0.07) and were also more likely to have visited a dentist within the past year. Mean PPD, CAL, and tooth loss were significantly higher among individuals with fewer than one dental visit per year compared with those who attended dental visits more regularly (*p* < 0.001). Multivariable logistic regression analyses demonstrated reduced odds of moderate-to-severe periodontitis among individuals with undiagnosed or diagnosed CD compared with healthy participants, although these associations did not reach statistical significance.

Kustro et al. [68], in a study comprising 50 participants with periodontitis who were also diagnosed with either CD or NCGS by a gastroenterologist, found greater periodontal pocket depths in the CD group (CD group: PPD = 3.25 ± 1.71 mm, whereas in the NCGS group: PPD = 2.88 ± 1.64 mm); however, this difference was not statistically significant. The mean papillary–marginal–alveolar index (PMA) in patients in the CD group was 28.9 ± 15.0%, compared with 24.88 ± 14.1% in the NCGS group. No statistically significant difference was found between the two study groups. The Fedorov–Volodkina hygiene index was 1.48 ± 0.3 in patients with CD and 1.51 ± 0.2 in patients with NCGS. All patients presented good oral hygiene. The mean value of the modified papillary bleeding index (MPBI) in patients with celiac disease was 0.21 ± 0.25, and in patients with NCGS, 0.24 ± 0.34. No statistically significant difference was found between the groups. Overall, no statistically significant differences in clinical periodontal parameters were reported between groups.

In the cross-sectional study of Nota et al. [69], based on structured self-reported questionnaires, 237 CD patients provided information regarding their oral hygiene habits, the presence of gingival signs and symptoms, the occurrence of gingival bleeding after toothbrushing, and the frequency of dental visits; however, statistically significant results were reported only for gingival bleeding in relation to GFD adherence. Among individuals participating in this study, 211 individuals (89%) reported strict adherence to a total GFD, 25 subjects (10%) reported partial adherence to a GFD, and one participant (0,42%) did not follow any type of diet. In patients adhering to a total GFD, gingival bleeding was reported by more than half of the participants, with occasional bleeding and absence of bleeding occurring at comparable frequencies, while a smaller subgroup reported persistent bleeding. By contrast, partial adherence to a GFD was more frequently associated with the absence of gingival bleeding, whereas occasional bleeding was reported by fewer participants, and persistent bleeding was limited to only a small minority. The single participant not adhering to any diet reported the presence of gingival bleeding. Thus, in this cross-sectional study, varying levels of adherence to a GFD in patients with CD are significantly associated with differences in self-reported gingival bleeding (*p* = 0.039).

Madi et al. [70], in a cross-sectional observational trial including 23 CD patients on a GFD and 23 healthy controls, reported that 83% of the control group, compared with only 30% of the CD group, were diagnosed with periodontitis. Diagnosis was made according to the Tonetti et al. criteria [71]. Periodontal parameters, including minimum and maximum PPD, minimum and maximum CAL, GI, and BoP were significantly higher in the control group compared to the CD group (*p* < 0.001). Within the control group, patients with periodontitis showed significantly higher GI (*p* = 0.007) and BoP (*p* = 0.001) compared with CD patients who also had periodontitis. It is also important to note that PI was negatively correlated with IgA (r = −0.460, *p* = 0.011), and similarly, GI and BoP were inversely and significantly correlated with IgA (r = −0.396, *p* = 0.003). Following multiple linear regression analysis, all periodontal parameters, including GI score, minimum and maximum PD, minimum and maximum CAL, and BOP, showed a significant but inverse association with CD.

In a more recent study, Sabancı et al. [72] compared clinical periodontal indices, such as PPD, PI, GI, and BoP, between 30 patients diagnosed with CD and 30 healthy controls. CD patients were diagnosed by medical professionals based on clinical symptoms, positive serological markers (anti-tTG and/or anti-EMA antibodies), and confirmation by a small intestinal biopsy, and they were all following a GFD. None of the participants had periodontitis. After a full-mouth periodontal examination, mean PPD in the CD group was 1.83 ± 0.48 mm, while in the healthy group it was 1.79 ± 0.47; PI was found to be 1.16 ± 0.34 in the CD group and 0.87 ± 0.66 in the control group; GI was 0.94 ± 0.57 in the CD and 0.92 ± 0.83 in the healthy group; and BoP was found to be 19.47 ± 18.99% and 19.28 ± 26.56% in the CD and control groups, respectively. However, these differences did not reach statistical significance. The percentage of participants with gingivitis was the same in the CD and the control group.

Overall, studies evaluating PPD and CAL generally reported similar clinical findings in adults with CD and healthy controls. Gingival inflammation and plaque accumulation were comparable between groups, while a few studies suggested slightly lower periodontal inflammation and periodontitis prevalence among CD patients. Observed differences were consistently modest and often associated with oral hygiene behaviors and dental attendance patterns. The main characteristics and findings of the studies evaluating the clinical periodontal and oral hygiene parameters in adults diagnosed with gluten-related disorders are summarized in Table 1.

#### 3.1.2. Children

In a cross-sectional observational study of 75 children, 52 children diagnosed with CD and 23 healthy controls, Mina et al. [73] assessed oral hygiene status and gingival inflammation using the simplified oral hygiene index (OHI-S) and GI, respectively. CD was confirmed by intestinal biopsy, while the absence of CD in healthy controls was verified through negative serological testing. Oral hygiene and gingival health were overall better in children with CD than in healthy controls; however, these differences did not reach statistical significance.

Shteyer et al. [74], in a cross-sectional observational study comprising 90 children, evaluated PI and tooth brushing frequency after dividing the sample into three groups: patients with newly diagnosed CD, CD patients receiving a GFD, and healthy controls. The authors reported statistically significant differences (*p* = 0.02) in PI among the three groups. The highest PI values were found in the newly diagnosed group, while the lowest values were recorded in the GFD-treated group. A significant difference in toothbrushing habits was observed between these groups; 30% of newly diagnosed children did not brush their teeth at all, compared with 0% in the celiac-treated group and 6.7% among healthy controls. In contrast, 60% of healthy children and 66.7% of celiac-treated children brushed their teeth twice a day, whereas only 26.7% of newly diagnosed children reported the same habit (*p* = 0.001).

Tsami et al. [75], in a cross-sectional observational study of 36 children diagnosed with CD of varying disease duration and adherence to a GFD, assessed oral hygiene status based on self-reported oral hygiene behaviors and frequency of dental visits and periodontal treatment needs using clinical indices, including GI, hygiene index (HI), and the periodontal screening and recording (PSR) system. The authors reported that periodontal treatment needs and oral hygiene status were primarily associated with oral hygiene practices and the presence of coexisting systemic conditions, such as type 1 diabetes mellitus or autoimmune thyroid disease, whereas overall periodontal status did not differ substantially from that observed in the general pediatric population.

Dababneh and Hijazeen [76] evaluated a sample of 86 children and adolescents, including 43 patients diagnosed with CD and 43 healthy controls. Among participants with CD, adherence to a GFD was documented; however, its potential impact on periodontal parameters and periodontal treatment needs was not assessed. Self-reported oral hygiene behaviors indicated a significantly lower frequency of tooth brushing among celiac patients compared with healthy controls (*p* < 0.05). Clinical periodontal assessment, including Plaque Index (PI), Gingival Index (GI), and Community Periodontal Index of Treatment Needs (CPITN), demonstrated significantly higher plaque scores in the CD group (*p* < 0.05), whereas no statistically significant differences were observed in the gingival index scores between groups. Shallow and deep periodontal pockets were more frequently observed among celiac patients, while overall CPITN scores did not differ significantly between the two groups, despite higher mean values in the CD group.

In a retrospective case-control study including 104 children with CD and 104 healthy controls, Alsadat et al. [77] reported that a significantly higher percentage of children with CD brushed their teeth compared with controls (*p* = 0.037). Brushing frequency was higher among children affected by the disease; however, this difference did not reach statistical significance.

In a trial comparing the oral hygiene of 62 children diagnosed with CD with 64 controls, Elbek-Cubukcu et al. [78] found that OHI-S was slightly higher in the CD group; however, this difference did not reach statistical significance. Notably, within the CD group, when comparing children adhering to a GFD with those not following one, oral hygiene was significantly better among children following the diet, as reflected by lower OHI-S values.

Bulut et al. [79] compared PI, GI, toothbrushing habits and frequency of dental visits between 52 children previously diagnosed with CD for more than six months and 52 patients recently diagnosed. Previously diagnosed children attended dental visits significantly more frequently than recently diagnosed patients (*p* = 0.039), while no statistically significant differences were observed in toothbrushing frequency, plaque or gingival indices between the two groups.

Collectively, GI values in children with CD were generally comparable to those observed in healthy controls. Similarly, periodontal screening indices (PSR, CPITN) did not demonstrate consistent differences between groups. In contrast, findings related to plaque accumulation were more variable across studies and were mainly associated with behavioral factors, particularly toothbrushing frequency and dental attendance patterns. Children adhering to a GFD often demonstrated better oral hygiene behaviors than newly diagnosed patients, although these differences did not consistently translate into clinically meaningful periodontal differences. The main characteristics and findings of the studies evaluating the clinical periodontal and oral hygiene parameters in children diagnosed with gluten-related disorders are summarized in Table 2.

### 3.2. Radiographic Findings of Alveolar Bone Loss Associated with Periodontitis

Few studies have investigated radiographic alveolar bone loss associated with periodontitis in patients diagnosed with CD and gluten-related disorders. In a trial comprising 25 patients with CD and 25 patients with NCGS, all additionally diagnosed with periodontitis, Kustro et al. [68] reported no statistically significant differences in clinical periodontal parameters between the two groups. Radiographic evaluation of mandibular bone mineral density (BMD) using orthopantomography and computed tomography revealed no significant differences between patients with CD and NCGS (mean BMD values: 1200–1300 HU and 1100–1250 HU, respectively).

In a cross-sectional analysis of HUNT4 2017–2019 data, Stødle et al. [80] evaluated the association between previously undiagnosed celiac disease and radiographically assessed periodontal bone loss. CD was defined hierarchically as (i) anti-tTG (TG2) IgA/IgG seropositivity, (ii) additional histological confirmation with duodenal biopsy showing Marsh grade 3a–c, and (iii) clinical relevance defined by recommendation of a GFD based on symptoms and repeated seropositivity. Panoramic radiographs were available for 485 seropositive participants, of whom 455 underwent biopsy, 307 had Marsh grade 3 histology, and 284 were recommended a GFD. Corresponding reference groups increased accordingly depending on the exposure definition. Periodontal bone loss was quantified radiographically as the percentage distance from the cementoenamel junction (CEJ) to the alveolar crest (AC) relative to root length and categorized as <15%, 15–33%, or >33% according to the 2017 classification [71]. Mean bone loss was lower in seropositive individuals compared with seronegative controls (15.9 ± 14.7% and 18.3 ± 15.4%, respectively, *p* < 0.001). Across all exposure definitions, CD was associated with a significantly lower likelihood of bone loss ≥15% (serology: PR 0.89, 95% CI 0.84–0.96; Marsh 3: PR 0.89, 95% CI 0.82–0.98; recommended GFD: PR 0.91, 95% CI 0.83–1.00), while no significant associations were observed for severe bone loss (>33%), suggesting a reduced prevalence of mild to moderate periodontal bone loss in untreated, undiagnosed CD.

Overall, available studies assessing radiographic findings of alveolar bone loss in patients with gluten-related disorders reported inconsistent results. While one study found no significant differences in BMD between patients with CD and NCGS, another trial suggested a lower prevalence of radiographic alveolar bone loss among untreated individuals with previously undiagnosed CD. Nevertheless, the available evidence remains extremely scarce.

The main characteristics and findings of the studies evaluating radiographic findings of alveolar bone loss associated with periodontitis in patients diagnosed with gluten-related disorders are summarized in Table 3.

### 3.3. Biochemical and/or Immunological Parameters Related to Periodontal Inflammation

#### 3.3.1. Salivary Antimicrobial Peptides

To date, limited evidence is available regarding the expression of antimicrobial peptides involved in host defense mechanisms against periodontal pathogens in patients with periodontitis and gluten-related disorders. Kustro et al. [81], in a cross-sectional study comparing four groups (patients with CD and periodontitis, patients with NCGS and periodontitis, patients with periodontitis without gluten intolerance, and healthy controls), reported significantly reduced oral fluid levels of LL-37 and HNP1–3 in generalized periodontitis associated with gluten-related disorders (CD/NCGS) compared with healthy controls. No significant differences were observed between the CD and NCGS groups (LL-37, ng/mL: CD with generalized periodontitis: 0.66 ± 0.19; NCGS with generalized periodontitis: 0.55 ± 0.18; generalized periodontitis without gluten intolerance: 0.81 ± 0.08; healthy controls: 0.98 ± 0.03; overall *p* ≤ 0.05, HNP1–3, ng/mL: CD with generalized periodontitis: 5.87 ± 0.91; NCGS with generalized periodontitis: 4.68 ± 0.28; generalized periodontitis without gluten intolerance: 6.14 ± 1.01; healthy controls: 7.31 ± 0.18; overall *p* ≤ 0.05). Notably, statistically significant positive correlations were reported between LL-37 and *P. gingivalis* (r ≈ 0.93, *p* ≤ 0.05) and between HNP1–3 and red-complex pathogens (r ≈ 0.67, *p* ≤ 0.05), suggesting an association between alterations in innate antimicrobial defense and periodontal pathogen burden in the context of gluten intolerance.

#### 3.3.2. Salivary and GCF Inflammatory Biomarkers

A study conducted by Madi et al. [70], including 23 CD patients on a GFD and 23 healthy controls, reported that CD patients presented a lower prevalence and severity of periodontitis compared with healthy controls, reflected by significantly lower periodontal clinical parameters and an inverse association between periodontal inflammation and IgA levels. In this trial, salivary concentrations of IL-17A, IL-1β, and IL-18 were also evaluated; however, no statistically significant differences in salivary IL-17A, IL-1β, or IL-18 levels were found between celiac patients on GFD and controls. Irrespective of celiac status, periodontitis was associated with higher salivary cytokine levels, with IL-1β being significantly elevated in control subjects with periodontitis (*p* = 0.035). Salivary biomarkers did not exhibit any significant or strong association with serological diagnostic tests. IL-17A showed negative correlations with anti-tTG antibodies and IgA, while IL-18 was negatively correlated with anti-EMA antibodies and IgA; similarly, IL-1β demonstrated a negative correlation with IgA. Furthermore, multiple linear regression analyses indicated that salivary cytokine levels were not significantly associated with disease status, in contrast to periodontal clinical parameters.

Sabancı et al. [72] compared clinical periodontal indices in 30 patients with CD and 30 healthy controls, none of whom had periodontitis, and found no statistically significant differences between the two groups. Salivary levels of inflammatory biomarkers, including TNF-α, Caspase-1 (Casp1), and Calcium (Ca), were also evaluated and did not differ significantly between patients with CD and healthy controls (TNF-α: 145.87 ± 29.95 and 195.24 ± 85.76 ng/mL, respectively, *p* = 0.161; caspase-1: 5.36 ± 1.54 and 6.68 ± 2.76 ng/mL, respectively, *p* = 0.362; calcium: 6.23 ± 2.60 and 7.24 ± 3.35 nmol/mL, respectively, *p* = 0.309). In addition, gingival crevicular fluid (GCF) levels of these biomarkers were assessed both as total amount and concentration. No statistically significant differences were observed between the groups in GCF total amounts (TNF-α: 199.50 ± 32.80 and 184.10 ± 28.57 ng/30 s, respectively, *p* = 0.103; caspase-1: 7.82 ± 1.16 and 7.19 ± 1.15 ng/30 s, respectively, *p* = 0.131; calcium: 12.98 ± 1.99 and 11.63 ± 2.13 nmol/30 s, respectively, *p* = 0.059), nor in GCF concentrations (TNF-α: 779.69 ± 435.20 and 725.42 ± 431.16 ng/μL, respectively, *p* = 0.702; caspase-1: 30.48 ± 16.17 and 28.38 ± 16.82 ng/μL, respectively, *p* = 0.563; calcium: 51.75 ± 27.26 and 46.37 ± 38.35 nmol/μL, respectively, *p* = 0.558). Overall, salivary and GCF levels of TNF-α, caspase-1, and calcium were comparable between CD patients adhering to a GFD and healthy controls.

Overall, studies evaluating antimicrobial peptides in patients with gluten-related disorders reported reduced salivary levels of LL-37 and HNP1–3 in individuals with periodontitis and gluten intolerance, indicating potential alterations in innate antimicrobial defense mechanisms. In contrast, studies assessing salivary and GCF inflammatory biomarkers (IL-17A, IL-1β, IL-18, TNF-α, Caspase-1, calcium) generally reported comparable levels between CD patients and healthy controls, with no consistent associations between these biomarkers and disease status. The main characteristics and findings of the studies evaluating biochemical and/or immunological parameters related to periodontal inflammation in patients diagnosed with gluten-related disorders are summarized in Table 4.

## 4. Discussion

This review investigated the impact of CD and NCGS on periodontal status by synthesizing data regarding clinical periodontal indices and oral hygiene parameters, radiographic findings, biochemical and immunological parameters related to periodontal inflammation in both adult and pediatric populations.

CD is a chronic, immune-mediated enteropathy with manifestations extending beyond the gastrointestinal tract and leading to multisystemic involvement and a broad range of extra-intestinal complications [1,2,5,8,14], including an established effect on oral health [59,60,61,62]. Persistent immune activation and dysregulation, chronic inflammation, nutrient deficiencies, xerostomia, and microbial dysbiosis found in CD and NCGS [12,13,36,40,41,50,57,58,65,74,78,82,83,84,85,86] constitute shared pathological findings, providing biological plausibility for an association with periodontitis [86,87,88,89,90,91,92]. More specifically, from an immunological perspective, gluten-related disorders are characterized by activation of both innate and adaptive immune pathways and increased production of pro-inflammatory cytokines, leading to systemic immune dysregulation and chronic inflammation [6,7,9,14,36,57,82,83,84,85]. Periodontitis is a chronic inflammatory disease, initiated and modulated by complex interactions between bacterial biofilm and the host’s immune response [71]. Therefore, the systemic inflammatory state observed in CD and NCGS may influence immune mechanisms in periodontal tissues and potentially contribute to periodontal inflammation [87,88,91]. In addition, nutrient deficiencies commonly associated with CD, including deficiencies in vitamin B complex, vitamins C and D, calcium, folate, and other micronutrients resulting from intestinal malabsorption and the inferior nutritional quality of gluten-free alternatives [12,13,32,33,34,35], may also affect periodontal health [92]. Periodontitis has also been increasingly linked to intestinal disorders through bidirectional pathogenic mechanisms, thereby further strengthening the biological rationale for a potential association with celiac disease [93,94,95]. However, the findings of the present review do not consistently support this hypothesis.

Findings from cross-sectional observational studies and self-reported questionnaires indicate that periodontal indices, including PPD, CAL, indices of gingival inflammation, plaque accumulation, and periodontitis prevalence, do not demonstrate consistent or statistically significant differences between individuals with CD and healthy controls or between CD and NCGS populations, irrespective of GFD status [65,66,68,69,70,72]. Sabancı et al. [72], in a sample comprising individuals without periodontitis, reported numerically higher PPD, GI, BoP, and PI values in CD patients compared with healthy controls. When comparing CD and NCGS patients with periodontitis, Kustro et al. [68] found greater PPD and PMA values in the CD group; however, these results did not reach statistical significance. Self-reported outcomes indicate a higher prevalence of perceived gingival problems among CD patients following a GFD [65,69], with evidence linking GFD adherence to compromised gingival health, despite similarities in oral hygiene and dental attendance patterns [65,69]. However, due to their subjective nature, these results present with limited reliability and interpretability. On the contrary, in the NHANES 2009–2012 data analysis, Spinell et al. [66] found significantly lower PPD values and a reduced proportion of sites with PPD ≥ 4 mm among individuals with CD, with no statistically significant differences detected in CAL or tooth loss. Reduced odds of moderate-to-severe periodontitis did not persist after multivariable adjustment. This study also demonstrated that CD patients reported more consistent oral hygiene practices, suggesting that the observed differences compared with healthy individuals were more closely associated with hygiene-related behaviors rather than reflecting a disease-specific biological effect. In the same direction, Madi et al. [70] reported significantly lower PPD, CAL, GI, and BoP values among CD patients adhering to a GFD, accompanied by reduced odds of periodontitis compared with healthy controls. Overall, most of these associations reported across studies lose statistical significance after multivariable adjustment and appear to be strongly influenced by behavioral confounders, such as oral hygiene practices and dental attendance patterns.

In pediatric populations, the available evidence similarly remains heterogeneous and largely inconclusive. Observed differences are mainly attributed to behavioral factors and oral hygiene parameters, reflecting increased awareness of health issues after CD diagnosis. Across all cross-sectional and case-control studies, pediatric patients with CD do not demonstrate worse periodontal status compared with healthy controls. Several studies even report comparable or better gingival health among CD patients, particularly those adhering to a GFD [73,76,77,78,79]. Newly diagnosed children with CD consistently exhibit poorer plaque control and less favorable toothbrushing habits compared with both GFD-treated children and healthy peers, whereas initiation and adherence to a GFD are repeatedly associated with improved oral hygiene behaviors and lower plaque accumulation [73,74,75,76,77,78]. Conversely, studies reporting higher plaque scores or increased periodontal treatment needs among children with CD rely on self-reported oral hygiene practices or mixed levels of GFD adherence. Some of these studies also include patients with coexisting systemic conditions, such as type 1 diabetes mellitus or autoimmune thyroid disease, which may act as primary determinants of observed periodontal findings [75,76]. Nevertheless, despite reported differences in plaque indices and gingival inflammation, periodontal treatment needs do not appear to differ substantially between groups. These results suggest that GFD adherence and health-related behaviors derived from early diagnosis may modulate oral hygiene patterns without translating into clinically important conclusions for managing the periodontal health of these children [73,75,77,79].

In CD, multiple mechanisms contribute to bone loss, which manifests as low BMD and early onset of osteoporosis [96]. In this context, alveolar bone loss related to periodontal inflammation in these patients was also assessed in the present review; However, the evidence is extremely limited and derives exclusively from two cross-sectional analyses, thereby limiting interpretability. Kustro et al. [81] reported no significant radiographic differences between patients with CD and NCGS presenting with established periodontitis, as mandibular BMD assessed by orthopantomography and computed tomography did not differ significantly between the two groups. On the contrary, population-based radiographic analyses by Stødle et al. [80] revealed a different pattern, with seropositive and biopsy-confirmed individuals exhibiting lower mean periodontal bone loss and a reduced likelihood of mild-to-moderate alveolar bone loss compared with seronegative controls. Notably, this apparent protective association was confined to early or moderate radiographic bone loss and did not extend to severe periodontal destruction, indicating that CD diagnosis and adherence to a GFD did not modify the risk of advanced alveolar bone loss. However, these findings should be interpreted cautiously, as they rely exclusively on radiographic, not clinical, periodontal assessment.

Evidence exploring biochemical and immunological markers of periodontal inflammation in the context of gluten-related disorders remains sparse and methodologically heterogeneous. With regard to innate antimicrobial defense and host defense mechanisms against periodontal pathogens, Kustro et al. [81] reported significantly reduced salivary levels of LL-37 and HNP1–3 in patients with generalized periodontitis and gluten intolerance, including both CD and NCGS, compared with healthy controls, while no differences were observed between CD and NCGS populations, indicating a shared defense mechanism. The same study identified strong positive correlations between antimicrobial peptide levels and periodontal pathogen burden, suggesting that alterations in host antimicrobial responses may accompany periodontal dysbiosis in the setting of gluten intolerance. In contrast, inflammatory cytokine profiling led to mainly neutral results. Madi et al. [70] evaluating CD patients adhering to a GFD, found no significant differences in salivary IL-17A, IL-1β, or IL-18 levels compared with healthy controls. Similarly, Sabancı et al. [72] reported comparable salivary and GCF levels of TNF-α, caspase-1, and calcium between CD patients on a GFD and healthy controls. Overall, current evidence suggests that, while antimicrobial peptide alterations may be observed in patients with gluten intolerance, salivary and GCF inflammatory biomarkers do not consistently reflect a CD-specific periodontal inflammatory profile, particularly in patients adhering to a GFD.

Despite the biologically plausible link between CD and periodontal inflammation, individuals with gluten intolerance do not appear to exhibit a uniformly worse periodontal status, nor is there sufficient evidence indicating a protective periodontal effect attributable to either condition. Observed differences, when present, tend to be modest and inconsistent across studies. They are also largely explained by behavioral factors, particularly oral hygiene practices and dental attendance patterns. In this context, both gluten-related disorders seem to act primarily as modifiers of health-related behaviors rather than as independent determinants of periodontal disease susceptibility or progression. Importantly, adherence to a GFD, while central to the clinical management of CD [1,2,25,26] and NCGS [40,41,42,43], although not universally efficient [27,28,29,30,31], does not appear to have a direct influence on periodontal health outcomes, but rather reflects increased disease awareness and lifestyle adjustments that may affect oral hygiene patterns. Moreover, several studies have highlighted the reduced nutritional quality of gluten-free products [12,35], which may result in significant micronutrient deficiencies, with potential implications for periodontal health [92]. Consequently, current evidence does not support the conclusion that individuals with CD or NCGS experience either systematically better or worse periodontal health compared with non-affected populations, nor does it support disease-specific periodontal management strategies beyond standard preventive and therapeutic approaches.

Overall, the scarcity of available evidence in combination with considerable methodological heterogeneity limits the interpretability and generalizability of our findings. Most studies rely on cross-sectional designs with relatively small patient samples. Additional heterogeneity is observed in participant characteristics, including age, disease duration, GFD adherence, and the inclusion of patients with different gluten-related disorder profiles. Variability is also evident in the diagnostic definitions of periodontal disease and the criteria used to assess periodontal outcomes, as different clinical thresholds were used across studies to define periodontitis. In addition, a considerable proportion of existing data is extracted from trials that lack comprehensive clinical evaluation and rely primarily on self-reported outcomes, thereby further compromising the reliability of the reported findings. Moreover, potential confounding factors, including oral hygiene practices, such as toothbrushing frequency and technique, oral health awareness and access to dental care, may also have influenced the reported outcomes and contributed to heterogeneity across studies. Hence, well-designed longitudinal studies with larger sample sizes and the use of standardized diagnostic criteria for both periodontal disease and gluten-related disorders are required to clarify whether CD or NCGS are associated with periodontal health outcomes.

## 5. Conclusions

Given the multisystemic effect of CD and NCGS, together with gluten-related disorders and other intestinal diseases’ well-documented impact on oral health, a biologically plausible link with periodontal inflammation can be hypothesized. However, evidence on this topic remains scarce and is characterized by considerable methodological variability, resulting in inconclusive findings. Further well-designed longitudinal studies are needed to determine the potential association between gluten intolerance and periodontal inflammation.

## Figures and Tables

**Table 1:** Summary of descriptive characteristics of studies evaluating clinical periodontal and oral hygiene parameters in adults diagnosed with gluten-related disorders.

Study/ Country	Study Design	Study Population, n	Age (Years)	Gluten-Related Disorders Profile	Diagnostic Criteria	Clinical Periodontal and Oral Hygiene Parameters Assessed	Main Conclusion
van Gils et al., 2017 [65] Netherlands	Cross-sectional observational study based on structure questionnaires	980 participants: 740 CD patients, 270 healthy patients	CD group: 55 (39–68) Control group: 53 (39–63)	Diagnosed CD GFD compliance/partial compliance: 91%/9%	Small intestine biopsy and/or positive serology	dental visits frequency, gingival problems (self-reported)	Patients with CD report a higher prevalence of gingival problems than healthy controls, despite similar dental visit frequency.
Spinell et al., 2018 [66] United States	Cross-sectional observational study	49 CD participants: 15 patients with diagnosed CD, 34 patients with undiagnosed CD	52 ± 14	Diagnosed CD on GFD Undiagnosed CD	Diagnosed CD: self-reported physician diagnosis while on a GFD, (+) anti-tTG, (+) anti-EMA antibodies Undiagnosed CD: (+) anti-tTG, (+) anti-EMA antibodies without self-reported diagnosis	PPD, CAL, tooth loss, periodontitis prevalence (CPP/ACP criteria [67]), toothbrushing frequency, dental visits frequency	CD is associated with lower PPD levels; however, no significant associations were observed regarding AL or periodontitis prevalence. Individuals with diagnosed CD reported more frequent dental visits and tended to exhibit better oral hygiene behaviors.
Kustro et al., 2020 [68] Ukraine	Cross-sectional observational study	50 participants: 25 patients with CD and periodontitis, 25 patients with NCGS and periodontitis	CD group: 41.03 ± 8.3 NCGS group: 40.38 ±8.1	Diagnosed CD Diagnosed NCGS GFD status not defined	Diagnosis made by a gastroenterologist	PPD, MPBI, Fedorov-Volodkina hygiene index, PMA	CD is associated with slightly greater probing pocket depths; however, no statistically significant differences are observed in periodontal clinical parameters between CD and NCGS groups.
Nota et al., 2020 [69] Italy	Cross-sectional observational study based on structure questionnaires	237 CD participants	15+	Self-reported CD total/partial/no- GFD compliance: 89%/10%/0,42%	Not defined	gingival bleeding (self-reported)	Varying levels of adherence to a GFD in patients with CD are associated with differences in self-reported gingival bleeding.
Madi et al., 2024 [70] Saudi Arabia	Cross-sectional observational study	46 participants: 23 CD patients on a GFD, 23 healthy patients	CD group: not reported Control group: 41	Diagnosed CD with GFD compliance > 1 year	Physician-confirmed diagnosis based on: (+) anti-tTG, and/or (+) anti-EMA antibodies	PPD, CAL, PI, GI, BoP, periodontitis prevalence [71]	CD patients on a GFD show a lower prevalence and severity of periodontitis compared with healthy controls, reflected by significantly lower periodontal clinical parameters and an inverse association between periodontal inflammation and IgA levels.
Sabancı et al., 2025 [72] Turkey	Cross-sectional observational study	60 participants without periodontitis (periodontal health and gingivitis): 30 patients with CD on a GFD, 30 healthy controls	CD group: 33.50 ± 6.28 Control group: 32.73 ± 5.70	CD GFD compliance	Diagnosis made by medical professionals based on: clinical symptoms, (+) anti-tTG IgA and/or (+) anti-EMA antibodies), small intestinal biopsy	PPD, PI, GI, BoP	No statistically significant differences in clinical periodontal parameters or gingivitis prevalence are observed between patients with CD on a GFD and healthy controls.

CD, celiac disease; NCGS, non-celiac gluten sensitivity; GFD, gluten-free diet; anti-tTG antibodies, anti-tissue transglutaminase antibodies; anti-EMA, anti-endomysial antibodies; PPD, probing pocket depth; CAL, clinical attachment level; PI, plaque index; GI, gingival index; BoP, bleeding on probing; MPBI, modified papillary bleeding index; PMA, papillary marginal alveolar index.

**Table 2:** Summary of descriptive characteristics of studies evaluating clinical periodontal and oral hygiene parameters in children with gluten-related disorders.

Study/ Country	Study Design	Study Population, n	Age (Years)	Gluten-Related Disorders Profile	Diagnostic Criteria	Clinical Periodontal and Oral Hygiene Parameters Assessed	Main Conclusion
Mina et al., 2008 [73] Argentina	Cross-sectional observational study	75 participants: 52 children with CD, 23 healthy children	4–12	Diagnosed CD GFD status not defined	CD was diagnosed by the gastroenterology specialist physician by means of intestinal biopsy as grade III or IV Absence of CD was confirmed by negative serological examinations (−) anti-tTG, (−) anti-EMA antibodies	GI, OHI-S	Oral hygiene and gingival health are overall better in children with CD than in healthy controls, although no statistically significant differences were observed.
Shteyer et al., 2013 [74] Israel	Cross-sectional observational study	90 participants: 30 patients with newly diagnosed CD, 30 CD patients on a GFD, 30 healthy controls	Newly diagnosed CD group: 6.9 ± 4.1 CD on a GFD group: 9.5 ± 4.5 Healthy controls: 6 ± 2.9	Newly diagnosed CD Diagnosed CD with GFD compliance > 6 months	gastroscopy and duodenal biopsy	PI, toothbrushing frequency	Newly diagnosed CD is associated with higher PI values and poorer toothbrushing habits, whereas children with CD on a GFD present lower PI values and better toothbrushing habits, comparable to healthy controls.
Tsami et al., 2010 [75] Greece	Cross-sectional observational study	35 CD participants	4–18	Pediatric CD GFD compliance	Not defined	GI, PSR, HI, toothbrushing frequency, dental visits frequency	In children and adolescents with CD, periodontal treatment needs and oral hygiene status are associated with oral hygiene behaviors and the presence of coexisting medical conditions, while overall periodontal status does not differ substantially from that of the general pediatric population.
Dababneh & Hijazeen et al., 2014 [76] Jordan	Prospective case-control study	86 participants: 43 CD patients, 43 healthy patients	CD group: 13.2 ± 2.85 Control group: 13.4 ± 2.74	Pediatric CD GFD compliance/non-compliance: 65.2%/34.8%	Not defined	GI, PI, CPITN, toothbrushing frequency	Children and adolescents with CD exhibit poorer oral hygiene and higher plaque accumulation compared with healthy controls, while no statistically significant differences were observed in gingival inflammation and overall periodontal treatment needs.
Alsadat et al., 2021 [77] Saudi Arabia	Retrospective case–control observational study	208 participants: 104 CD patients, 104 healthy patients	CD group: 10.67 ± 2.39 Control group: 10.69 ± 2.36	Diagnosed CD GFD status not defined	Small intestine biopsy	toothbrushing frequency	Although children with CD are more likely to brush their teeth than healthy controls, brushing frequency does not differ significantly between groups.
Elbek-Cubukcu et al., 2023 [78] Turkey	Cross-sectional observational study	126 participants: 62 CD patients, 64 healthy controls	CD group: 12.31 ± 3.43 Control group: 12.12 ± 3.3	Diagnosed pediatric CD GFD compliance not reported	(+) anti-EMA antibodies, small-bowel biopsy	OHI-S	Children with CD show slightly better oral hygiene than healthy controls without reaching statistical significance, while within the CD, adherence to a GFD was associated with significantly better oral hygiene.
Bulut et al., 2023 [79] Turkey	Cross-sectional observational study	78 CD participants: 52 previously diagnosed patients, 26 recently diagnosed patients	Previously diagnosed CD group: 9.35 ± 2.98 Recently diagnosed CD group: 8.12 ± 3.23	Diagnosed CD on a GFD > 6 months, Recently diagnosed CD	(+) anti-tTG, antibodies, small-bowel biopsy	GI, PI, toothbrushing frequency, dental visits frequency	Children previously diagnosed with CD attended dental visits significantly more frequently than recently diagnosed patients, while no statistically significant differences were observed in toothbrushing frequency, plaque or gingival indices between the two groups.

CD, celiac disease; GFD, gluten-free diet; anti-tTG antibodies, anti-tissue transglutaminase antibodies; anti-EMA, anti-endomysial antibodies; OHI-S, simplified oral hygiene index; GI, gingival index; PI, plaque index; HI, hygiene index; CPITN, community periodontal index of treatment needs; PSR, periodontal screening and recording.

**Table 3:** Summary of descriptive characteristics of studies evaluating radiographic findings of alveolar bone loss associated with periodontitis in patients diagnosed with gluten-related disorders.

Study/ Country	Study Design	Study Population, n	Age (Years)	Gluten-Related Disorders Profile	Diagnostic Criteria	Radiographic Findings of Alveolar Bone Loss Associated with Periodontitis	Main Conclusion
Kustro et al., 2020 [68] Ukraine	Cross-sectional observational study	50 participants: 25 patients with CD and periodontitis, 25 patients with NCGS and periodontitis	CD group: 41.03 ± 8.3 NCGS group: 40.38 ± 8.1	Diagnosed CD Diagnosed NCGS GFD status not defined	Diagnosis made by a gastroenterologist	BMD	Mandibular BMD does not differ significantly between patients with CD and those with NCGS.
Stødle et al., 2024 [80] Norway	Cross-sectional observational study	5212/5182/5184 participants: 485/307/284 patients with previously undiagnosed CD, 4727/4875/4900 healthy controls (diagnosis based on positive serology/+biopsy confirmation/+GFD recommendation)	(+) anti-tTG previously undiagnosed CD group: 53.0 ± 15.6 Control group: 51.5 ± 16.4	Previously undiagnosed, biopsy-confirmed CD (Marsh grade 3a–c); GFD recommended in a subset of patients	(+) anti-tTG (IgA/IgG) antibodies, duodenal biopsy confirmation (Marsh grade 3a-c), GFD recommendation based on clinical assessment and repeated seropositivity	Radiographic periodontal bone loss (<15%, ≥15–33%, >33% of root length) corresponding to the stages of severity according to the 2017 classification [71]	Previously undiagnosed CD is consistently associated with a lower likelihood of radiographic periodontal bone loss ≥15%, irrespective of the diagnostic definition applied (serology, Marsh grade 3, or recommended GFD), with no significant association observed for severe bone loss (>33%).

CD, celiac disease; NCGS, non-celiac gluten sensitivity; GFD, gluten-free diet; anti-tTG antibodies, anti-tissue transglutaminase antibodies; IgA, Immunoglobulin A; IgG, Immunoglobulin G; BMD, bone mineral density.

**Table 4:** Summary of descriptive characteristics of studies evaluating biochemical and/or immunological parameters related to periodontal inflammation in patients diagnosed with gluten-related disorders.

Study/ Country	Study Design	Study Population, n	Age (Years)	Gluten-Related Disorders Profile	Diagnostic Criteria	Biochemical and/or Immunological Parameters Related to Periodontal Inflammation	Main Conclusion
Kustro et al., 2021 [81] Ukraine	Cross-sectional observational study	120 participants: 30 patients with CD and periodontitis, 30 patients with NCGS and periodontitis, 30 patients with periodontitis, 30 healthy controls	28.03 ± 3.3	Diagnosed CD Diagnosed NCGS GFD status not defined	Diagnosis made by a gastroenterologist	Salivary antimicrobial peptides: LL-37 (cathelicidins), HNP1–3 (α–defensins)	Patients with periodontitis and gluten intolerance exhibit reduced salivary levels of antimicrobial peptides (LL-37 and HNP1–3), which are positively correlated with periodontal pathogen burden, suggesting an altered innate antimicrobial defense.
Madi et al., 2024 [70] Saudi Arabia	Cross-sectional observational study	46 participants: 23 CD patients on a GFD, 23 healthy patients	CD group: not reported Control group: 41	Diagnosed CD with GFD compliance > 1 year	Physician-confirmed diagnosis based on: (+) anti-tTG, and/or (+) anti-EMA antibodies	Salivary pro-inflammatory cytokines IL-17A, IL-1β, IL-18	In CD patients adhering to a GFD, salivary pro-inflammatory cytokine levels (IL-17A, IL-1β, and IL-18) do not differ significantly from healthy controls and are not independently associated with disease status, indicating that salivary interleukins have limited value in reflecting the impact of CD on periodontal inflammation.
Sabancı et al., 2025 [72] Turkey	Cross-sectional observational study	60 participants without periodontitis (periodontal health and gingivitis): 30 patients with CD on a GFD, 30 healthy controls	CD group: 33.50± 6.28 Control group: 32.73 ±5.70	CD GFD compliance	Diagnosis made by medical professionals based on: clinical symptoms, (+) anti-tTG IgA and/or (+) anti-EMA antibodies), small intestinal biopsy	Salivary and GCF Inflammatory biomarkers TNF-*α*, Ca, and Casp1	No statistically significant differences were observed in salivary or GCF levels of TNF-α, Ca, or Casp-1 between CD patients adhering to a GFD and healthy controls.

CD, celiac disease; NCGS, non-celiac gluten sensitivity; GFD, gluten-free diet; anti-tTG antibodies, anti-tissue transglutaminase antibodies; anti-EMA, anti-endomysial antibodies; LL-37, Leucine–Leucine-37; HNP1–3, Human Neutrophil Peptides 1–3; IL-17A, Interleukin-17A; IL-1β, Interleukin-1 beta; IL-18, Interleukin-18; GCF, gingival crevicular fluid; TNF-α, Tumor Necrosis Factor-alpha; Ca, calcium; Casp-1, Caspase-1.

## Data Availability

No new data were created or analyzed in this study. Data sharing is not applicable to this article.

## Supplementary Materials

The following supporting information can be downloaded at: https://www.mdpi.com/article/10.3390/jcm15082828/s1, Table S1: Detailed search strategy for electronic databases, Table S2: Excluded articles and reasons for exclusion [97,98,99,100,101,102,103,104].

## Abbreviations

The following abbreviations are used in this manuscript:

CD	Celiac disease
NCGS	Non-celiac gluten sensitivity
GFD	Gluten-free diet
anti-tTG	anti-tissue transglutaminase
anti-EMA	anti-endomysial
PPD	Probing pocket depth
CAL	Clinical attachment level
PI	Plaque index
BoP	Bleeding on probing
GI	Gingival index
MPBI	Modified papillary bleeding index
PMA	Papillary marginal alveolar index
OHI-S	Simplified oral hygiene index
CPITN	Community periodontal index of treatment needs
PSR	Periodontal screening and recording
BMD	Bone mineral density
LL-37	Leucine-leucine-37
HNP1–3	Human Neutrophil Peptides 1-3
IL-17A	Interleukin-17A
IL-1β	Interleukin-1 beta
IL-18	Interleukin-18
GCF	Gingival crevicular fluid
TNF-α	Tumor Necrosis Factor-alpha
Ca	Calcium
Casp-1	Caspase-1
