# Supplementary material for: Periodontal Status of Patients with Celiac Disease and Non-Celiac Gluten Sensitivity: A Literature Review
Source: J Clin Med. 2026 Apr 8;15(8):2828. doi: 10.3390/jcm15082828 (PMC13115811; doi:10.3390/jcm15082828)
Supplement: Supplementary file 1 [file jcm-15-02828-s001.zip › jcm-4192015-supplementary.pdf]

Periodontal Status of Patients with Celiac Disease and Non-Celiac Gluten Sensitivity: A Literature Review

Thaleia Angelopoulou [1] and Yiorgos A. Bobetsis[2]

[1] DDS, Postgraduate Student, School of Medicine, National and Kapodistrian University of Athens, Greece; thangelop@yahoo.gr

[2] Associate Professor, Department of Periodontology, School of Dentistry, National and Kapodistrian University of Athens, Greece; ybobetsi@dent.uoa.gr

* Correspondence: ybobetsi@dent.uoa.gr; Tel.: +306936613292

Supporting Material

Table S1. Detailed search strategy for electronic databases.

Electronic databases

Database	Search (January 10, 2026)	Studies
**PubMed**	("Periodontitis"[MeSH] OR "Periodontal Diseases"[MeSH] OR "Dental Scaling"[MeSH] OR "Debridement"[MeSH] OR "non-surgical periodontal therapy"[tiab] OR "periodontal therapy"[tiab] OR "periodontal treatment"[tiab] OR "periodontitis"[tiab] OR Gingivitis[MeSH] OR "Gingival Diseases"[MeSH] OR "gingivitis"[tiab] OR SRP[tiab] OR "Dental Plaque"[MeSH] OR "Oral Hygiene"[MeSH] OR "Oral Manifestations"[MeSH] OR "oral manifestations"[tiab] OR "Dental Health Services"[MeSH] OR "oral health"[tiab] OR xerostomia[tiab] OR "saliva"[tiab] OR "salivary"[tiab] OR "gingival crevicular fluid"[tiab] OR GCF[tiab] OR "periodontal bone loss"[tiab] OR "oral microbial profiles"[tiab] OR "oral ecosystem"[tiab] OR "salivary microbiota"[tiab] OR "oral microbiome"[tiab] OR	238

	"oral status"[tiab]) AND ("Celiac Disease"[MeSH] OR "celiac disease"[tiab] OR "coeliac disease"[tiab] OR NCGS[tiab] OR "non-celiac gluten sensitivity"[tiab] OR "gluten sensitivity"[tiab] OR Gluten[MeSH] OR gluten[tiab] OR "gluten-related disorders"[tiab] OR "gluten-free diet"[tiab] OR "gluten free diet"[tiab] OR GFD[tiab] OR "marsh types"[tiab])	
**Web of Science**	(Periodontitis OR Periodontal Diseases OR Dental Scaling OR Debridement OR non-surgical periodontal therapy OR periodontal therapy OR periodontal treatment OR periodontitis OR Gingivitis OR Gingival Diseases OR SRP OR Dental Plaque OR Oral Hygiene OR Oral Manifestations OR oral manifestations OR Dental Health Services OR oral health OR xerostomia OR saliva OR salivary OR gingival crevicular fluid OR GCF OR periodontal bone loss OR oral microbial profiles OR oral ecosystem OR salivary microbiota OR oral microbiome OR oral status) AND (Celiac Disease OR celiac disease OR coeliac disease OR NCGS OR non-celiac gluten sensitivity OR gluten sensitivity OR Gluten OR gluten OR gluten-related disorders OR gluten-free diet OR gluten free diet OR GFD OR marsh types)	660
**Scopus**	TITLE-ABS-KEY ("periodontitis" OR "periodontal disease" OR "periodontal diseases" OR "non-surgical periodontal therapy" OR "periodontal therapy" OR "periodontal treatment" OR "scaling and root planing" OR "dental scaling" OR "debridement" OR "gingivitis" OR "gingival diseases" OR "SRP" OR "dental plaque" OR "oral hygiene" OR "oral health" OR "oral manifestations" OR "xerostomia" OR "saliva" OR "salivary" OR "gingival crevicular fluid" OR "GCF" OR "periodontal bone loss" OR "oral microbial profiles" OR "oral ecosystem" OR "salivary microbiota" OR "oral microbiome" OR "oral status") AND ("celiac disease" OR "coeliac disease" OR "NCGS" OR "non-celiac gluten sensitivity" OR "gluten sensitivity" OR "gluten"	528

	OR "gluten-related disorders" OR "gluten free diet" OR "gluten-free diet" OR "GFD" OR "marsh types")	
**Cochrane Library**	(periodontitis OR periodontal disease OR periodontal diseases OR nonsurgical periodontal therapy OR non-surgical periodontal therapy OR periodontal therapy OR periodontal treatment OR scaling and root planing OR dental scaling OR debridement OR gingivitis OR gingival disease OR gingival diseases OR oral health OR xerostomia OR saliva OR salivary OR gingival crevicular fluid OR periodontal bone loss) AND (celiac disease OR coeliac disease OR celiac OR coeliac OR non celiac gluten sensitivity OR non-celiac gluten sensitivity OR gluten sensitivity OR gluten OR gluten related disorders OR gluten free diet OR gluten-free diet OR GFD OR marsh types)	69

Grey Literature

**BASE**	("periodontitis" OR "periodontal diseases" OR "gingivitis" OR "gingival diseases" OR "non-surgical periodontal therapy" OR "periodontal therapy" OR "periodontal treatment" OR "dental scaling" OR "debridement" OR "scaling and root planing" OR SRP OR "periodontal bone loss" OR "dental plaque" OR "oral hygiene" OR "oral health" OR "oral manifestations" OR xerostomia OR saliva OR salivary OR "gingival crevicular fluid" OR GCF OR "oral microbial profiles" OR "oral ecosystem" OR "salivary microbiota" OR "oral microbiome" OR "oral status" OR "dental health services") AND ("celiac disease" OR "coeliac disease" OR NCGS OR "non-celiac gluten sensitivity" OR "gluten sensitivity" OR gluten OR "gluten-related disorders" OR "gluten-free diet" OR "gluten free diet" OR GFD OR "marsh classification" OR "marsh types")	961

**ProQuest**	NOFT((“periodontitis” OR "periodontal diseases" OR gingivitis OR "gingival diseases" OR "non-surgical periodontal therapy" OR "periodontal therapy" OR "periodontal treatment" OR "dental scaling" OR debridement OR "scaling and root planing" OR SRP OR "periodontal bone loss" OR "dental plaque" OR "oral hygiene" OR "oral health" OR "oral manifestations" OR xerostomia OR saliva OR salivary OR "gingival crevicular fluid" OR GCF OR "oral microbial profiles" OR "oral ecosystem" OR "salivary microbiota" OR "oral microbiome" OR "oral status" OR "dental health services") AND ("celiac disease" OR "coeliac disease" OR NCGS OR "non-celiac gluten sensitivity" OR "gluten sensitivity" OR gluten OR "gluten-related disorders" OR "gluten-free diet" OR "gluten free diet" OR GFD OR "marsh classification" OR "marsh types"))	42
**Google Scholar**	("periodontitis" OR "periodontal diseases" OR "gingivitis" OR "gingival diseases" OR "non-surgical periodontal therapy" OR "periodontal therapy" OR "periodontal treatment" OR "dental scaling" OR "debridement" OR "scaling and root planing") AND ("celiac disease" OR "coeliac disease" OR "non-celiac gluten sensitivity" OR NCGS OR "gluten sensitivity")	100
**Research Gate**	“periodontitis” “periodontal diseases” “gingivitis” “celiac disease” “non-celiac gluten sensitivity”	100

Table S2 Excluded articles and reasons for exclusion

Records identified from Electronic Databases	
Full-text articles excluded, with reasons (n=10)	
Author/Year	Reason for exclusion
Fernandez-Feo et al., 2013 [97]	1
Dane and Gürbüz, 2016 [98]	1
Tian et al., 2017 [99]	1
Cruz et al., 2018 [60]	1

Shahraki et al., 2019 [100]	1
Panelli et al., 2020 [101]	1
Ahmed et al., 2021 [102]	1
Moreau et al., 2021 [103]	1
Acar et al., 2025 [85]	1
Lenander-Lumikari et al., 2000 [104]	2

Reasons for exclusion:

1 - Different clinical outcomes assessed (n= 9)

2 - Different study design (n= 1)
